# Supplementary material for: First insights into the microbiome of Tunisian Hyalomma ticks gained through next-generation sequencing with a special focus on H. scupense
Source: PLoS One. 2022 May 19;17(5):e0268172. doi: 10.1371/journal.pone.0268172 (PMC9119559; doi:10.1371/journal.pone.0268172)
Supplement: Attachment — Submitted filename: Comments annotated_complete.pdf [file pone.0268172.s002.pdf]

First insights into the microbiome of Tunisian *Hyalomma* ticks gained through next-generation sequencing with a special focus on *H. scupense*

Hayet Benyedem[1,2], Abdelmalek Lekired[3], Moez Mhadhbi[1], Mokhtar Dhibi[1], Rihab Romdhane[1], Soufiene Chaari[4], Mourad Rekik[5], Hadda-Imene Ouzari[3], Tarek Hajji[6] and Mohamed Aziz Darghouth[1]

[1] Laboratoire de Parasitologie, Institution de la Recherche et de l'Enseignement Supérieur Agricoles and Univ. Manouba, École Nationale de Médecine Vétérinaire de Sidi Thabet, Sidi Thabet, Tunisia

[2] Faculty of Sciences of Tunis, University of Tunis El Manar, Tunis, Tunisia

[3] Université Tunis El Manar, Faculté des Sciences de Tunis, Laboratoire des Microorganismes et Biomolécules Actives (LR03ES03), Tunis, Tunisia

[4] Laboratoire pharmaceutique vétérinaire MEDIVET, Soliman, Tunisia

[5] International Centre for Agricultural Research in the Dry Areas (ICARDA), Tunis, Tunisia

[6] Univ. Manouba, Higher Institute of Biotechnology - Sidi Thabet, Laboratory of Biotechnology and Valorization of Bio-Geo-Resources (LR11ES31), Ariana, Tunisia

* Corresponding author: darghouth@iresa.tn (MAD)

Abstract

Ticks are one of the most important vectors of several pathogens affecting humans and animals. In addition to pathogens, ticks carry diverse microbiota of symbiotic and commensal microorganisms. In this study, we have studied the first Tunisian insight into the microbial composition and microbiota diversity, richness, and their variation between the most dominant *Hyalomma* species infesting Tunisian cattle, male and female as well as between different life stages of *H. scupense*. In this regard, Next Generation sequencing for the 16S rRNA (V3-V4 region) and metagenomic analysis were established. the analysis of the bacterial diversity reveals that *H. marginatum* and *H. excavatum* have greater diversity than *H. scupense*. Furthermore, microbial diversity and composition vary according to the tick's life stage and sex in the specific case of *H. scupense*. The endosymbionts *Francisella*, *Canditatus* Midichloria, and *Rickettsia* were shown to be the most prevalent in *Hyalomma*. *Rickettsia*, *Francisella*, *Ehrlichia*, and *Erwinia* are the most common zoonotic bacteria found in *Hyalomma* ticks.

According to our findings, all studied *Hyalomma* tick species possess zoonotic bacteria genera and could potentially operate as vectors for various zoonoses.

Keywords: microbiome, Next-Generation sequencing, Hyalomma, endosymbionts, pathogens, Tunisia.

Introduction

Ticks are strict hematophagous ectoparasites of vertebrate animals and humans. Distributed all over the world, they are implicated in the transmission of several pathogens (viruses, bacteria, protozoa, and helminths) of medical and veterinary importance. Accordingly, ticks are considered to be the first world wild vectors of animal diseases and the second of human diseases behind mosquitoes [1,2].

Livestock farming is one of the main agricultural sectors in North African countries and Tunisia [3]. Ticks are widespread in Tunisia over different geographical regions, *Hyalomma spp* is the most prevalent genus infesting cattle [4,5].

Tick infestation represents a major threat in cattle breeding due to their capacity to transmit three major tick-borne pathogens (TBPs), and to induce different diseases, specifically, anaplasmosis, babesiosis, and theileriosis [4,6,7]. Tropical theileriosis, which is caused by the protozoan *Theileria annulata* and transmitted by *Hyalomma scupense*, is the major tick-borne disease affecting North African and Tunisian cattle [8–10].

Besides pathogens, ticks are also harboring a large variety of symbiotic and commensal microorganisms [11]. Symbiotic bacteria play important roles in tick survival particularly via the synthesis of some essential vitamins and cofactors, especially vitamins of group B, which are lacking in the highly specialized hematophagous tick diet [12–14]. They are also implicated in tick adaptation to environmental stress, and finally, they are essential for tick's reproduction fitness [11,12]. These symbiotic bacteria can also influence the colonization, maintenance, and transmission of pathogens [15,16]. Ticks carry at least 10 different genera of maternally inherited endosymbiotic bacteria [12,13]. Three of these endosymbionts namely, *Coxiella*, *Francisella* and *Midichloria* are specific to ticks. *Coxiella* is the most frequent endosymbiont, it is present in two-thirds of tick species [12,17]. *Midichloria* which infect the mitochondria has been reported in some tick species, while *Francisella* has only been detected in few tick species [18]. Indeed, *Francisella*, *Coxiella*, *Rickettsia* endosymbionts are obligate nutritional symbiotic bacteria that have conserved all the major genes encoding for vitamins B synthesis despite

their highly reduced genome [19–21]. For instance, *Coxiella* endosymbiont is carrying the genes encoding for vitamins B7 (biotin), B2 (riboflavin), B9 (folic acid), and their cofactors [22,23]. In the *Rickettsia* endosymbionts genome, Hunter's team confirmed the presence of all genes encoding for the biosynthesis of folic acid [21]. Also, *Francisella* endosymbiont genome is including all the genetic pathways for the synthesis of folic acid, biotin, and riboflavin [16,24].

The importance of B vitamins in tick survival and development has been established by Duron and al. in 2018 with *Francisella* in the soft tick *Ornithodoros moubata* [13]. An experimental elimination of the symbiont using antibiotic treatment was followed by a reduced tick survival and the appearance of some physical abnormalities, interestingly, these effects were fully restored with a supplement of vitamin B. Similar experiments with *Coxiella* in *Amblyomma americanum*, *Haemaphysalis longicornis*, and *Rhipicephalus microplus* resulted in lower reproductive fitness and prevented even the development into adults in *R. microplus*. While, being one of the maternally transmitted endosymbiotic, the role of *Canditatus Midichloria mitochondrii* in their host biology has not been well established. However, some studies have suggested their role in molting and tick blood meal metabolism following the increase of this symbiont after feeding [25,26].

Taken altogether the above general data are highlighting the important role of obligate symbionts in tick biology and reproduction, opening, accordingly, a potential window of opportunities for developing prototypes of innovative and environmentally friendly control measures targeting these symbionts.

In North Africa and Tunisia, the majority of studies on livestock and zoonotic tick-borne pathogens have focused essentially on pathogens and diseases identification, and their epidemiology and control using conventional approaches. However, almost there is no data on microbiota and endosymbionts of the most dominant and important Tunisian tick species of the *Hyalomma* genus. In this context, our work intends to provide the first comprehensive study carried out on major *Hyalomma* species in Tunisia and more generally in North Africa, for describing, firstly, the microbial diversity and richness of tick microbiota in *Hyalomma* ticks infecting livestock in different bioclimatic and geographic regions in Tunisia, and establishing secondly, the prevalence of their endosymbiotic bacteria. We aim in the present work to focus more, and for the first time to our knowledge, on the symbionts associated with the different life stages of the monotropic *H. scupense* cattle tick, the vector of tropical theileriosis, and to carry out a comparative study with the adult stages of other dominant cattle *Hyalomma* tick species in Tunisia.

Too much use of "firstly" & "secondly"

Material and methods

Study area and tick sampling

A total of 776 adults ticks were sampled during June, July, and August 2020. Adult ticks were collected from cattle in 6 different bioclimatic stages and 9 Tunisian administrative governorates to explore the whole microbial diversity of Tunisian ticks and to investigate their endosymbionts (Fig 1). For this purpose, cattle barns were recruited according to the presence of the previous history of tropical theileriosis cases to secure the presence of significant infestation prevalence with *Hyalomma* ticks and in particular *H. scupense* (Table 1 and Fig 1). Furthermore, engorged *H. scupense* nymphs were collected from walls of some cattle breeding between October and November 2020 from El Hissiene site (upper semi-arid stage, Ariana, Tunisia).

Fig 1. Tunisia map showing the sampling sites.

Tick species identification

Tick species were first identified morphologically according to the key of Walker et al [27]. Subsequently, a molecular characterization was done using rRNA 16S and rRNA 12S genes for all species and in particular on fully engorged females and nymphs. All sequences were compared to sequences in GenBank using BLAST analysis. We have considered that ticks of the same species are sharing a similarity of ≥97% [28]. All DNA samples were stored at -20°C until their use.

Genomic DNA extraction and pooling

We have first sterilized the exoskeleton of ticks to eliminate surface contaminants using bleach at 1% for 30 seconds followed by three washes with ultra-pure water according to the protocol of Binetruy et al [29]. DNA was extracted from individual adult ticks using the DNeasy Blood &Tissue Kit (QIAGEN, Valencia, CA), while DNA extraction from engorged nymphs and eggs was performed following the Wizard Genomics DNA kit (Promega, Madisson) protocol. The extracted DNA was stored at -20°C for future use.

To study the specific microbial population of ticks, DNA from individual samples was pooled together in equal concentrations for each species and stage. This approach was used in this first investigation to reduce the financial and technical burden of the NGS analysis.

A total of six pools were prepared for next-generation sequencing composed as follow: 60 nymphs of *H. scupense*, 4 pools of 120 adult ticks composed each of, 30 *H. scupense* females, 30 *H. scupense* males, 30 *H. marginatum adults*, 30 *H. excavatum adults*, furthermore one pool of ticks' eggs was also included in the analysis.

Library preparation for Metagenomic sequencing

DNA was quantified using the Qubit dsDNA HS Assay kit (Invitrogen). Library preparation was carried out using NextEra cd index prep on Illumina Miseq. Briefly, 12.5 ng of DNA from each sample were amplified using a v3-v4 primer of 16s rDNA. Samples were dual indexed the pooled and quantified as the final library. An amount of 4 nM of the pooled library was normalized and denatured with NaOH, then samples were loaded on the Illumina Miseq instrument for sequencing.

Metagenomic analysis

Metagenomic analysis was carried out using qiime2 (Quantitative Insights Into Microbial Ecology) [30] (version 2020.6) from (https://qiime2.org/), raw reads were filtered and adapters were trimmed, reads were denoised using deblur and the table of features was generated, the subsampling of features for the alpha diversity analysis was carried out after the rarefaction of samples by 5624 features. BarPlots of relative abundance were created using the Phyloseq package and Vagans library on Rstudio and the Piecharts were created with Plotly after treatment with Biom algorithm.

Prevalence and transmission of symbionts

Independent *Francisella* and *Rickettsia* endosymbionts identification was performed using PCR by amplifying the rRNA 16S and glta gene with specific primers (Table 2). PCR conditions started with denaturation step at 94°C for 7 min followed by 40 cycles at 94°C for 45s, 62°C for 30s, and 72°C for 45s, with a final extension at 72°C

for 7 min. The negative DNA template for symbiont was verified systemically by the amplification of ticks' 18S rRNA gene using universal primers of eucaryotic. PCR products of glta and 16S were purified and sequenced from both directions. Sequences were verified using BLAST.

Maternal transmission assay

A total of nine fully engorged females of *H. scupense, H. excavatum* and *H. marginatum* were collected live from cattle. Engorged ticks were separately incubated at 27°C and 90% relative humidity until the end of oviposition. DNA was extracted from 300 eggs of each tick and stored at -20°C until further use.

Table 1. Bioclimatic stage, governorate, delegation, farms visited, surveyed cattle and ticks collected.

Bioclimatic stage	Governorate	Delegation	Farms	Cattle	Ticks
Upper semi-arid	Bizerte	Besbesia	3	68	79
Upper semi-arid	Ariana	Hessiènne	4	36	56
Subhumid	Bizerte	El Alia	1	7	20
Subhumid	Bizerte	Aousseja	1	16	33
Upper semi-arid	Seliana	El krib	5	40	45
Sub-humid	Bizerte	Ghezala	7	36	151
Humid to sub-humid	Jendouba	Fernena	7	56	32
Humid to sub-humid	Beja	Amdoun	4	25	27
Humid to sub-humid	Beja	Beja	3	10	7
Humid	Beja	Nefza	4	32	34
Upper Arid	Kairouan	El Ala	8	30	65
Upper Arid	Kasserine	Sebitla	7	35	53
Semi-arid	Zaghouan	Fahs	9	42	13
Lower semi-arid	Sousse	Kalaa Kbira	11	32	30
Humid	Jendouba	Tabarka	8	25	131

Table 2. Targeted genes, primers, and nucleotide sequences per tick species.

Species	Target gene	Primers	Nucleotide sequence (5′–3′)	Reference
Ref. [31] *Rickettsia* endosymbiont	Citrate synthase (gltA)	gltA-F	TCCTACATGCCGACCATGAG	[31]
		gltA-R	AAAGGGTTAGCTCCGGATGAG	
Francisella endosymbiont	16S rRNA	Fran16S-F	CAGGACTAGCTTATAGTTGCTGG	[19]
		Fra16S-R	CATCTGCGACAGCCTAAAAGGC	
Ref.[32] *Theileria annulata*	Cytochrome b	Cyto F	CAGGGCTTTAACCTACAAATTAAC	[32]
		Cyto R	CCCCTCCACTAAGCGTCTTTCGACAC	

Results

Tick species identification

Due to the sampling design *Hyalomma scupense* was the main collected species, representing more than P1,L2 40.0% of collected ticks (n = 776), followed by *Hyalomma marginatum* (17.80%) then *Hyalomma excavatum* (17.36%), *Haemaphysalis punctata* (18.81%), *Rhipiciphalus spp.* (6.94%) and finally *Hyalomma impeltatum* P1,L4 (0.14%). Morphologically identified specimens of *H. scupense*, marginatum, and excavatum used in our study P1,L5 were then confirmed with molecular identification and sequencing using rRNA 16S and rRNA 12S genes.

Microbial diversity in ticks

We have essentially focused on the characterization of the microbial composition and microbiota diversity, richness, and their variation between male and female as well as between different life stages of *H. scupense*. P1,L3 We have also carried out a comparative study with *H. marginatum* and *H. excavatum* which are commonly infesting cattle in Tunisia at the adult stage. In this regard, a 16S rRNA (V3-V4 region) sequencing and metagenomic analysis were established.

Denoising reads with deblurring were generated 97081 features where the greatest number of reads was observed in female *H. scupense* and the lowest number of reads was detected in male *H. scupense*. The denoising P2,L3 allowed the selection of 124 representative sequences (OTU) with a mean length of 148 bp. The taxonomic assignment of OTUs was carried out using the Silva 16S database with 99% of cut-off, the classifier was trained using the qiime feature-classifier algorithm for V3-V4 primers.

Scanned with CamScanner

P3 Bacteria profiling from different identified livestock species showed that proteobacteria were the dominant phylum with more than 97% of relative percent abundance (RPA), followed by Actinobacteria, Firmicutes, and Deinococcota. Overall, 16 bacterial families were detected, among those, Rickettsiacea, Francisellacea, Enterobacteriaceae, Bacillaceae, and Brevibacillacea were the most predominant families in the examined tick species (Fig 2). In the male of *H. scupense*, Enterobacteriaceae was the most prevalent bacterial family with 69.5% RPA followed by Rickettsiaceae 22.9% and Francisellacea 2.56%. However, *Francisella* was the dominant family in the female of *H. scupense* with 78.8% of RPA followed by Rickettsiaceae with 19.9% of RPA. In the two other *Hyalomma* species, Rickettsiaceae were the most abundant followed by Francisellacea.

Fig 2. Family level taxonomic composition of *Hyalomma excavatum, H. marginatum* and *H. scupense* adult tick.

Three symbiotic bacterial genera were detected in the analyzed ticks, these were *Francisella, Rickettsia*, and *Midichloria mitochondrii*. *Francisella* and *Rickettsia* genera were detected in all three species of *adult Hyalomma*,
P4,L3 they were also present in all the stages of *H. scupense* (Fig 3), however at different rates. The highest RPA of *Francisella* was recorded in the females of *H. scupense* with an RPA of 78.8%, followed by *H. marginatum* sample with an RPA of 37.9%. However, the lowest rate was observed in the male of *H. scupense*. The Rickettsia genera
P4,L6 make up the vast majority of *H. excavatum* with 70.8% RPA, it was also detected at a relatively high abundance of 34.5% in *H. marginatum*. *Midichloria mitochondrii* was highly present *in H. marginatum and H. excavatum* with an RPA of 34.5 and 17.3% respectively.

Fig 3. Relative abundance (%) of bacterial genera according to tick species and life stage of *Hyalomma scupense*. Organisms listed in the legend appear from top to bottom in order of highest relative abundance.

We have detected also some genus of pathogen bacteria for instance *Ehrlichia, Erwinia, Dietzia, Escherichia, Shigella, Pseudomonas*, as well as the two other symbiotic/pathogenic bacteria: *Francisella* and *Rickettsia*. To examine the possible difference of bacterial population structure between species, sexes, and life stages, alpha diversity parameters were calculated (Table 3 and Fig 4).

Table 3. Alpha diversity indexes.

Scanned with CamScanner

Sample	Obs	Chao1	se.chao1	ACE	se.ACE	Shannon	Simp	InvSimp	Fisher
H. scupese female	12	12,00	0,24	12,46	1,73	0,96	0,47	1,87	1,61
H. scupense male	15	15,00	0,00	15,00	1,55	1,12	0,57	2,35	2,08
H. scupense nymph	23	23,25	0,73	23,67	2,29	1,23	0,55	2,25	3,43
H. excavatum	15	15,00	0,00	15,00	1,93	1,29	0,67	3,00	2,08
H. marginatum	15	15,00	0,00	15,00	1,93	1,65	0,76	4,16	2,08
H. scupense eggs	10	10,50	1,28	11,79	1,37	1,40	0,70	3,37	1,30

Fig 4. Alpha diversity and richness parametres of Hyalomma scupense microbiota. a: Shannon alpha diversity between species; c: Chao alpha diversity between species; b: Chao alpha diversity between life stages.

Notably, there was a significant difference between adult species, *H. excavatum H. marginatum* samples have a significantly more diverse microbiome than *H. scupense* as indicated by the Chao and Simpson indexes (Table 3 and Fig 3). *H. scupense,* female ticks have less microbial diversity than males, furthermore, there was a significant difference between the relative abundance of the bacterial genus and species detected in female versus male ticks, indeed, 40 species were detected in males while 35 species were recorded in females (Fig 5). Indeed, *Francisella* endosymbionts constituted by far a dominant percentage of the microbiome of female ticks comparatively to male ticks, 78.8% and 2.51% respectively. Although, there was a significant difference between different stages since adult ticks exhibit a greater extent of microbial diversity. We then examined the microbial richness using the Shannon diversity index (Table 3), and similar trends were observed as with the Chao index because *H. scupense* samples have less rich diversity than the other two species. However, in the case of *H. scupense*, nymphs were by far the most reach in diversity relative to the two other life stages.

Fig 5. Genus's level taxonomic composition of male and female *Hyalomma scupense* ticks.

Maternal transmission

To test whether *Francisella* and *Rickettsia* endosymbionts have a maternal transmission. Samples of eggs of nine fully engorged female of females of *H. scupense, H. excavatum* and *H. marginatum* ticks were screened by

Scanned with CamScanner

PCR amplification. PCR product was sequenced and revealed that all the pools of eggs of each female were positive for the two symbionts with an overall transovarial transmission efficiency of 100%. Although using NGS, we ~~have~~ detected the presence of these two symbionts in *H. scupense* eggs at an RPA of 16% and 18.1% for *Francisella* and *Rickettsia* respectively. However, the most dominant endosymbiont detected in *H. scupense* eggs was *Midichloria mitochondrii* with 65.5% RPA (Fig 3). This endosymbiont was not detected in *H. scupense* adult ticks, while as reported ~~early~~ it was highly ~~re~~presented in adults of *H. marginatum* and to a lesser extent in of *H. excavatum*.

Prevalence of symbionts

P1,L2-4

To assess the prevalence of symbionts in *H. scupense* tick nymphs and adults, as well as the effect of gender, we first sequenced the PCR product for each symbiont to validate the sequences of the symbionts. A total of 60 engorged nymphs were tested and the results show that 45% of them carry *Francisella* endosymbiont while 22.22 % of them harbor *Ricketessia* endosymbiont for male ticks, PCR amplification of 40 males *H. scupense* revealed that 35.13% harbor *Farncisella* endosymbiont. Interestingly, 80% of half-engorged females were positive for *Francisella* endosymbiont while 66,66% of them were positive with Rickettsia endosymbiont,

P1,L7 suggesting the potential reproductive role of those symbionts and their correlation with the tick stage.

Discussion

Previous surveys on ticks microbiota have shown carriage of multiple symbionts, which are very important for their physiology, fecundity, and vector competence [33–35]. Currently, several studies were carried out on

P1,L3 ticks microbial population of *Ixodes, Rhipiciphalus, Haemaphysalis, Amblyomma, Dermacentor* [36–42].

However, little is known about the ~~tick diversity~~ microbiome of the *Hyalomma* ~~genus~~ associated with cattle. Moreover, there is no information regarding the microbial population structure of the most dominant Tunisian

P1,L6 ~~tick cattle~~ *H. scupense* despite ~~their~~ veterinary and economic importance. We present here the first

P1,L7 characterization of the whole microbial communities of the monotropic tick cattle *H. scupense* according to the

Scanned with CamScanner

stages and sex and its comparison with the microbial diversity of adults *H. scupense* tick of two other dominant *Hyalomma* species which adult stages are infesting cattle, namely *H. excavatum* and *H. marginatum*.

P2,L1 Next generation sequencing of the hypervariable V3-V4 bacterial 16S rRNA gene was performed on *Hyalomma* specimens with Illumina Miseq. Similar to other studies, we found that the most dominant phylum was those belong to Proteobacteria. Additionally, the bacterial microbiota of *Hyalomma* was dominated by three principal genera, namely the two symbiotic/pathogenic bacteria *Francisella* and *Rickettsia*, and the endosymbiotic *Candidatus midichloria*. Previous studies on other genera like *Rhipicephalus* have shown that they were mainly infected with *Coxiella* and *Rickettsia* with an association of *Coxiella*-like endosymbionts with females [38,43]. A more recent study on the microbiome of *Hyalomma* species infesting livestock in Pakistan showed that *Francisella, Rickettsia,* and *Coxiella* were the most dominant tick bacterial identified species [44,45].
P2,L9-10 Nevertheless, our results correlate with another survey in the Turkish tick population where they don't detect *Coxiella* in their *Hyalomma* samples but they found that *Francisella* and *Rickettsia* predominate in the microbiome of *H. excavatum* and *H. marginatum* [28]. In our study, it was not entirely surprising that we detected the genus of *Francisella* at high relative abundance associated with the absence of the acquisition of
P2,L13 *Coxiella* endosymbiont, despite their higher rate of transovarial transmission and their abondance in vast other tick genera. *Coxiella* endosymbiont was detected using NGS in a large variety of hard ticks including *Amblyomma americanum* [46], *Haemaphysalis longicornis* [36], *Ornithodoros amblus* [47], *Rhipicephalus microplus* [48], and *Rhipicephalus sanguineus* [38]. Our findings are similar to those of Duron research team who lack to find or
P2,L16 detected at low frequency *Coxiella* endosymbiont in approximately one-third of the targeted species [48]. Furthermore, Gehrart and his team suggested that *Francisella* has replaced Coxiella in the Gulf Coast tick, *Amblyomma maculatum* [49].

We suggested that the lack of detection of *Coxiella* infection in all our samples ahead of the abundance of another maternally inherited endosymbiont may be due to the replacement and competition of this ancient endosymbiont with other maternally inherited endosymbionts. In our case, we have revealed the presence of three other genera of maternally inherited bacteria: *Francisella, Candidatus midichloria,* and *Rickettsia* endosymbiont in all tick specimens. The high distribution among all our tick species of *Francisella* suggests that they may have independently replaced *Coxiella* endosymbiont as an alternative obligate symbiont. The
P3,L8-9 importance of *Francisella* in Tunisian *Hyalomma* ticks is probably reflecting the particularities of the microbiome community interactions and its long coevolution with their host ticks through the ecological specialization of

Scanned with CamScanner

Rephrase sentence or shorten. Explain or omit

P3,L10 these ticks to their restricted diet and niches. Although it is important to emphasize that physiological and nutritional investigation on the symbiotic functions of *Francisella* are required to validate their role as alternative

vague term

P3,L12 obligate symbionts. Moreover, Genome data of these bacteria demonstrate that they evolved by developing adaptative mechanisms implicated in tick survival, indeed, they have highly conserved the major genes responsible for the synthesis of vitamins B, cofactors, and some amino acids like Coxiella endosymbiont despite their restraint genome [19–21,24,49].

P4 Interestingly, we report here, using high throughput sequencing, the first detection of the obligate

reorder words

P4,L2 mitochondrial intracellular tick endosymbiont *Candidatus Midichloria mitochondrii* in eggs of *Hyalomma* tick with a high abundance of more than 65%, ~~while~~ however this density was considerably decreased in the other stages of *H. scupense* (nymphs and adults), ~~simultaneously~~ along with the increase of ~~the~~ other two endosymbionts. The distribution and the transovarial transmission of this intracellular endosymbiont in *H. scupense* suggested that this association might be obligate, and have a potential role in the physiological fitness of *Hyalomma scupense* tick. Furthermore, this bacterium is significantly abundant in *H. marginatum* and to a lesser extent in *H. excavatum* with RPA of 34.5 and 17.8% respectively. Recently, Selmi ~~and~~ et al reported that *Candidatus Midichloria mitochondrii was* prevalent at a rate of 8% in *H. dromedarii* and *H. impeltatum* partially fed ticks collected from dromedaries in Tunisia [50]. Moreover, the detection of this endosymbiotic bacterium in other hard ticks is very

Relative abundance or what? okay?

choose one

P4,L11 variable or absent [26,51]. *Candidatus midichloria mitochondrii* is well-described in *I. ricinus* [25] where it was detected in the mitochondria of ovaries. These bacteria which are included in the α-Proteobacteria entered the mitochondria of the oocyst cells. About 94 -100 % of adult females of *I. ricinus* are infected with this bacterium with a 100 % transovarial transmission rate [25].

Additionally, we have studied various aspects of microbial diversity including species, sex, and life stage. The investigation of the microbial diversity with *Haylomma* species indicates that the adults of *H. marginatum* and *H. excavatum* are more diverse and richer than *H. scupense*. Previous microbial diversity studies in three *Hyalomma* species collected in Turkey indicated also that *H. excavatum* and *H. marginatum* are more diverse than *H. aegyptium* [28]. This diversity variation is influenced by multiple factors; mainly environmental factors like habitat [52], geographical dispersion [53], bioclimatic conditions [54], and the hosts the tick feed on [55]. *H. marginatum* and *H. excavatum* are probably more exposed to complex environmental conditions and pass

Rewrite to make it fit in

P5,L8 through different hosts during their development due to their polytropic life cycle and the diversity of their hosts at juvenile stages [56]. Moreover, it is well known that host blood meals have a strong impact and altered tick

⇒ Declutter the discussion by omitting abundance related redundancy
⇒ Discuss more about the probable underlying factors that shape the microbiome using more references.

Scanned with CamScanner

microbial diversity, composition, and species richness as mentioned by Swei and Kwan [39]. Ticks acquire more microorganisms including pathogens from hosts during their blood meals. The most diverse hosts and the largest number of vertebrates hypothetically drive to more diverse microbiota [57]. This is the case for the outdoor H. P5,L13 *marginatum and H. excavatum* ticks, indeed, these tick juveniles feed, according to their tropism, on a variety of small terrestrial mammals and birds, whilst their adults could engorge on a variety of large herbivores such as goats, sheep, cattle, horses, camels [58,59]. In contrast, *H. scupense* exhibits a domestic behavior, and cattle P5,L16 are by far the almost exclusive host for adults and juveniles in Tunisia [5,60]

Microbial composition was affected also by sex. Females *H. scupense* microbiota was less diverse and P6,L2 richness than males, correlated with the high relative abundance of endosymbionts than males especially their OTUs number. We found also a higher prevalence of *Francisella* in females and especially in engorged females P6,L4 (100%). Similar results were reported in other studies that typically [43,61,62] In the same way, Van Treuren and his team found that females show less diverse microbiota than males [53]. The increased RA of *Rickettsia* and *Francisella* endosymbiont that we recorded in females relative to males could be an adaptation to reproductive requirements and transovarial transmission. In addition, we demonstrated efficient maternal transmission of these two endosymbionts.

According to our metagenomic analysis, we found a significant loss of bacterial species richness in adults compared to nymphs. *Francisella* and *Ricketssia* become dominant at older life stages correlated with loss of microbiome species richness. Our results are similar to another study on the microbiome of *Ixodes pacificus* [39]. This change is obviously due to the variation of tick activity and their metabolic function between their different life stages and sex. Indeed, ticks obligatory endosymbionts have a crucial role in development, nutrition reproduction fitness, and tick adaptation to new environmental conditions in the tick life cycle [11,57]. Furthermore, it is also important to consider a potential role for the environment in the acquisition and P7,L8 expression of microbiota particularly for related species having distinct interaction with their environment due to the particularities of their life cycle and ecological niches like for instance the case of the domestic *H. scupense* with the outdoor ticks *H. marginatum* and *H. excavatum*.

P8 The significance of this finding for the ticks themselves and their hosts requires more investigation to be well understood, regarding their veterinary and zoonotic importance in particular for *Francisella*, and *Rickettsia* bacteria.

Scanned with CamScanner

Conclusion

Overall, the analysis of the bacterial diversity within three *Hyalomma* species reveals that *H. marginatum* and *H. excavatum* have greater diversity than *H. scupense*. Furthermore, microbial diversity and composition vary according to the tick's life stage and sex in the specific case of *H. scupense*. The endosymbionts *Francisella, Canditatus* Midichloria, and *Rickettsia* were shown to be the most prevalent in *Hyalomma*. According to our findings, all studied *Hyalomma* tick species possess zoonotic bacteria genera and could potentially operate as vectors for various zoonoses. *Rickettsia, Francisella*, and *Rickettsia* are the most common zoonotic bacteria found in *Hyalomma* ticks.

This study provides general information about microbial communities in *Haylomma* ticks and their endosymbionts. This information provides critical clues for future studies aimed at the prevention and control of neglected tick and tick-borne diseases in the area using innovative approaches.

Acknowledgments

This study was financially supported by the ICARDA - CGIAR Research Program on Livestock (CRP Livestock), and the "Laboratory of epidemiology of enzootic infections in herbivores in Tunisia: application to control" (LR16AGR01) funded by the Ministry of Higher Education and Scientific Research, Tunisia. This study is also carried out under the MOBIDOC scheme, funded by The Ministry of Higher Education and Scientific Research through the PromEssE project and managed by the ANPR

Competing interests

No competing interests have been declared by the authors.

Ethical statement

The animals used in this study belonged to farmers who consented to their sampling. The animals were gently restrained by their owner to collect ticks, in the same way as for a routine clinical [illegible]

Scanned with CamScanner
