# Supplementary material for: First insights into the microbiome of Tunisian Hyalomma ticks gained through next-generation sequencing with a special focus on H. scupense
Source: PLoS One. 2022 May 19;17(5):e0268172. doi: 10.1371/journal.pone.0268172 (PMC9119559; doi:10.1371/journal.pone.0268172)
Supplement: Attachment — Submitted filename: PONE-D-21-29206.docx [file pone.0268172.s001.docx]

**Dear Prof./ Editor-in-Chief of PLOS ONE,**

I hope this mail finds you well.

Thank you for your trust and efforts.

With pleasure I reviewed the manuscript PONE-D-21-29206 entitled “First insights into the microbiome of Tunisian *Hyalomma* ticks gained through next generation sequencing with a special focus on *H. scupense*”.

It is good manuscript, based on firm scientific background, and add-value to our knowledge on microbial composition harboured by these ticks.

**For the authors:**

Thank you for this research, which deals with very interesting subject with little data on ticks and its symbiotic and commensal/pathogenic bacteria.

I have some comments/remarks to the respected authors. As follow:

I- **Generally**:

- Please add abbreviation list for all abbreviations you used e.g. rRNA, 16S, 25S, V3-V4, OUT, RPA, NGS.
- '*Candidatus Midichloria mitochondrii*' please review this bacterium species scientific name and then after unify witting name and style of this bacterium throughout the manuscript.

II- **Other comments:** many typo errors, please revise the manuscript well

**Abstract:**

- **P7: next generation**

**The analysis…**

**….for 16S RNA of?????**

***Hyalomma* spp. or use *H. scupense***

**Common bacterial genera founf in *Hyalomma* ticks**

**Introduction**

- **P8 : *Hyalomma* spp. is the most prevalent ticks infesting cattle**

**Materials and methods**

- **P10: 'adult' not italic**

**Cattle breeding barns**

**16S and 25S rRNA genes (repeat in P13)**

**'bleach' please specify the material you used**

- **P11: 'tick's egg' replace by *Hyalomma* spp. egg**
- **P11 and P13: in table 2 mentioned that you used a specific primer to amplify *Cytochrome b* mtDNA gene of *Theileria annulata* from the collected ticks. Interestingly, I did not any results or discussion on the usage of this primer, why??!!, although in the introduction section and study design, you emphasized cattle theileriosis and ticks role in it also, mentioned that ticks were collected from sites with pervious history of theileriosis.**
- **P12: 9 instead of nine**

**Results**

- **P13: spp. not italic**
- **P14: However, Francisellacea was the dominant family in the female of *H. scupense* with 78.8% of…..**

**'adult' not italic**

**Fig.2: adult ticks**

- **P15: Fig 4. *Hyalomma scupense***
- **P16: '** **suggesting the potential reproductive role of those symbionts and their correlation with the tick stage. ' please omit or rephrase or remove to discussion section as no suggestions in the results. Results are based on facts not suggestions.**

**Discussion**

- **P18: Selmi et al. reported**
- **P19: 'RA'??!! you mean RPA or another meaning?**

Dear respected authors, thank you again.

Regards.
