# Supplementary material for: First insights into the microbiome of Tunisian Hyalomma ticks gained through next-generation sequencing with a special focus on H. scupense
Source: PLoS One. 2022 May 19;17(5):e0268172. doi: 10.1371/journal.pone.0268172 (PMC9119559; doi:10.1371/journal.pone.0268172)
Supplement: Attachment — Submitted filename: RESPONSE TO REVIEWERS.docx [file pone.0268172.s003.docx]

**RESPONSE TO REVIEWERS**

**PONE-D-21-21-29206**

**First insights into the microbiome of Tunisian *Hyalomma* ticks gained through next-generation sequencing with a special focus on *H. scupense***

Hayet Benyedem^1,2^, Abdelmalek Lekired^3^, Moez Mhadhbi^1^, Mokhtar Dhibi^1^, Rihab Romdhane^1^, Soufiene Chaari^4^, Mourad Rekik^5^, Hadda-Imene Ouzari^3^, Tarek Hajji^6^ and Mohamed Aziz Darghouth^1^

Corresponding author: darghouth@iresa.tn

Sidi Thabet, December 30, 2021

**Technical report**

Dear Editor,

Please note that figure 1 was removed.

Kindly find below the author’s answer, in blue, to all reviewer comments.

**Reviewer #1:**

I- General comments:

• Please add abbreviation list for all abbreviations you used e.g. rRNA, 16S, 25S, V3-V4, OUT, RPA, NGS.

The following abbreviation list was added, please refer to lines 361-369

**Abbreviation list:**

rRNA: Ribosomal ribonucleic acid

16S: Gene coding for the small ribosomal 16 RNA gene

12S: gene coding for the small ribosomal 12 RNA gene

V3-V4: the hypervariable regions of the 16S rRNA gene

OUT: Operational Taxonomic Unit

RPA: Relative Percent Abundance

NGS: Next Generation Sequencing

*M. mitochondrii: Midichloria mitochondrii*

• 'Candidatus Midichloria mitochondrii' please review this bacterium species scientific name and then after unify witting name and style of this bacterium throughout the manuscript.

Verified and standardized as *Midichloria mitochondrii* from NCBI Taxonomy Browser*,* all over the MS

II- Other comments: many typo errors, please revise the manuscript well

The manuscript was revised and typo errors were corrected

III- Specific comments

Abstract:

• Point 7 (P7): next generation

Corrected as required, please refer to page 2 line 3

The analysis…

corrected (T in capital), Page 2 Line 4

….for 16S RNA of?????

Corrected as follows: for the 16S rRNA (V3-V4 region) of tick bacterial microbiota and…, please refer to page 2 lines 3 and 4

Hyalomma spp. or use H. scupense

corrected as follows: *Hyalomma* spp, please refer to Page 2 line 8

Common bacterial genera found in Hyalomma ticks

corrected as follows: Accordingly, *Hyalomma* ticks could represent potential vectors for these zoonotic bacterial agents…, please refer to Page 2 lines 9-10

Introduction

• P8 : Hyalomma spp. are the most prevalent ticks infesting cattle

corrected as required, please refer to page 2 line 21

Materials and methods

• P10: 'adult' not italic

Corrected as required, all over the MS

Cattle breeding barns

Corrected, please refer to Page 5 line 1

16S and 25S rRNA genes (repeat in P13)

Corrected as follows: 16S and 12S rRNA genes, please refer to page 5 line 16 and all over the MS

'bleach' please specify the material you used

The used material was specified as required: commercial bleach, please refer to P5 line 22

• P11: 'tick's egg' replace by Hyalomma spp. Egg

The sentence was changed as follows: one pool of *H. scupense* eggs and…, please refer to p6 line 5

• P11 and P13: in table 2 mentioned that you used a specific primer to amplify Cytochrome b mtDNA gene of Theileria annulata from the collected ticks. Interestingly, I did not any results or discussion on the usage of this primer, why??!!, although in the introduction section and study design, you emphasized cattle theileriosis and ticks role in it also, mentioned that ticks were collected from sites with pervious history of theileriosis.

The authors agree with reviewer comment, the primers were removed from the table, ~~it was a careless mistake,~~ please refer to table 2

• P12: 9 instead of nine

Done, please refer to p7 line 9.

Results

• P13: spp. not italic

Corrected, please refer to p8 line 8

• P14: However, Francisellacea was the dominant family in the female of H. scupense with 78.8% of…..

Corrected, please refer to p9 line 16

'adult' not italic
Fig.2: adult ticks

Corrected

Please refer to p9 line 20

• P15: Fig 4. Hyalomma scupense.

corrected in italic, p11 line 9

• P16: ' suggesting the potential reproductive role of those symbionts and their correlation with the tick stage. ' please omit or rephrase or remove to discussion section as no suggestions in the results. Results are based on facts not suggestions.

The text was removed

Discussion

• P18: Selmi et al. reported

Corrected as required, please refer to p14 line 6

• P19: 'RA'??!! you mean RPA or another meaning?

Corrected: RPA, please refer to p15 line 27

**Reviewer #2:** Dear Authros, the article is written well but there are some shortcomings that can be incorporated to improve it

1. There are formatting errors (pdf file is attached)

All suggestions made by Reviewer #2 were considered. The changes in the MS were put in blue color.

For the Reviewer #2 questions hand written in the pdf version:

Q1: Rephrase, no sense (p4, Study area and tick sampling)

The text was modified as follows:

For this purpose, cattle barns were selected according to previous tropical theileriosis cases

Please refer to lines 24-27 p4.

Q2 Why not 18S rRNA, ticks are eukaryote?

We agree the Reviewer comment, ticks are eukaryotes of course, however, we targeted the mitochondrial 12S and 16S ribosomal RNA (rRNA) genes.

In fact, rRNA 16S and 12S rRNA are the most commonly used markers to study tick’s phylogeny and they have been demonstrated to be able to differentiate the species of some ticks (Anderson et al., 2004; Vial et al., 2006; Chen et al., 2012). Furthermore, the number of annotated sequences of rRNA 16S genes in GenBank was increasing recently providing a strong database for tick’s species.

The text was modified as follows:

…was done using the mitochondrial 16S and 12S rRNA genes…

Please refer to p5 line 16

Q2 : How 6 groups?

Four groups for adults, one for nymphs and one for eggs

In the MS, the sentence war modified as follows:

A total of six pools were prepared for next-generation sequencing composed as follows: one pool composed by 60 nymphs of *H. scupense*, one pool of *H. scupense* eggs and 4 pools of adult ticks composed each one of 30 *H. scupense* females, 30 *H. scupense* males, 30 *H. marginatum* adults and 30 *H. excavatum* adults. Please refer to p6 lines 4-7

Comment 2. There need statistical analysis like regression to find prevalence and correlation with relation to area, tick stage and gender of the tick

We added statistical analysis using chi 2 test to check the significance of the correlation between of symbiont prevalence and sex, and life stage of *H. scupense.*

We have studied the prevalence of symbiont only for *H. scupense* samples. In this study we targeted the symbionts of *H. scupence* ticks. Moreover, the parameter “Area” was not taken into consideration in the current study

The following text was added

To compare the prevalence of symbionts between sexe and life stage of *H. scupense* ticks, the test chi 2 was performed. Results were considered significant at 5% threshold.

Please refer to p7 lines 4 and 5

The “Result section” was also modified, and the test result was added. Please refer to p10 line 22 and p12 lines 3-4 .

3. There is a recent published article related to your article which should be incorporated in discussion

Al-Hosary A, Răileanu C, Tauchmann O, Fischer S, Nijhof AM, Silaghi C. Tick species identification and molecular detection of tick-borne pathogens in blood and ticks collected from cattle in Egypt. Ticks Tick Borne Dis. 2021 May;12(3):101676. doi: 10.1016/j.ttbdis.2021.101676. Epub 2021 Jan 26. PMID: 33540276.

We included this article to the discussion section. The following text was added:

Recent study on the detection of pathogens and *M. mitochodrii* in Egyptian cattle ticks using the Reverse Line Blot hybridization (RLB) showed that 11.6% of pools of H. excavatum are infected with *M. mitochondrii* while only 2.9% of *R. annulatus* pools were infected by this bacteria [50]. Please refer to p14 lines 8-12

The citation was added to the reference list at the end of the MS

4. There is need to change the figure 3 and it is suggested to keep the colours referring to each organism must be same in the whole figure.

The fig 3 was changed as required

Rephrase, omit the redundancy,

discuss more about the probable understanding factors that shape the microbial using more references

The discussion was partially rephrased and the following text was added:

This diversity is influenced by several determinants, mainly environmental factors like, habitat [52], geographical dispersion [53], bioclimatic conditions [54], sample location [55], soil and plant associated bacteria [56], and also the tick hosts [57,58].

*H. marginatum* and *H. excavatum* are probably exposed to more complex environmental conditions as they pass through different hosts during their development particularly at juvenile stages [59]. Moreover, it is well known that blood meals have a strong impact on tick microbial diversity, composition, and species richness as mentioned by Swei and Kwan [38]. Indeed, ticks acquire more microorganisms, including pathogens, from hosts during their blood meals. More diverse hosts and larger numbers of vertebrates hosts hypothetically drive to more diverse microbiota [60]. This is probably the case for the outdoor *H. marginatum* and *H. excavatum* ticks, indeed, these tick juveniles feed, according to their tropism, on a variety of small terrestrial mammals and birds, whilst their adults can engorge on a variety of large herbivores such as goats, sheep, cattle, horses, camels [61,62]. In contrast, *H. scupense*, exhibits a domestic behavior, and cattle are by far the almost exclusive host for adults and juveniles in Tunisia [5,63]. Other studies found that the use of different tick species feeding on the same host don’t even have the same microbiomes, suggesting therefore, that tick microbiome is, to a certain degree, governed by species specific determinants [64]. Please refer to p14 and 15 lines 21 – 9.

P8: Mention influence on tick physiology functional role of this endosymbiont for further insights, better to rewrite

The following text was added as suggested:

Our results on symbionts of Hyalomma ticks may contribute to improve our knowledge on the interaction between tick bacterial communities and their hosts opening the way to develop subsequently applied research targeting the development of new control options against ticks and tick-borne pathogens. Bacterial endosymbionts may affect tick’s physiology and their reproductive fitness, they may also influence tick’s vectorial capacity for transmitted pathogens, and finally they may interact with the tick hosts with potential veterinary and zoonotic outcomes in particular for Francisella and Rickettsia bacteria. Please refer to p16 lines 12 – 17.
