# Supplementary material for: First insights into the microbiome of Tunisian Hyalomma ticks gained through next-generation sequencing with a special focus on H. scupense
Source: PLoS One. 2022 May 19;17(5):e0268172. doi: 10.1371/journal.pone.0268172 (PMC9119559; doi:10.1371/journal.pone.0268172)
Supplement: Attachment — Submitted filename: Response to Reviewers.docx [file pone.0268172.s004.docx]

**Response to Reviewers**

Dear Editor,

Dear reviewers,

On behalf of my coauthors, I would like to thank you again for the revision processing of our manuscript PONE-D-21-29206 “First insights into the microbiome of Tunisian *Hyalomma* ticks gained through next-generation sequencing with a special focus on *H. scupense*”. We would like to thank the reviewers for their thoughtful comments and efforts towards improving our manuscript. In the following, we addressed comments to reviewer requirements below. In our response letter, the reviewers’ comments are numbered and colored in grey if no changes are needed and in black color if changes are required. The corresponding responses (prefaced by “Author response”) follow below, in blue color. Corresponding changes are red colored in the manuscript text on the revised file (Revised Manuscript with track changes).

1. If the authors have adequately addressed your comments raised in a previous round of review and you feel that this manuscript is now acceptable for publication, you may indicate that here to bypass the “Comments to the Author” section, enter your conflict of interest statement in the “Confidential to Editor” section, and submit your "Accept" recommendation.

Reviewer #2: All comments have been addressed

2. Is the manuscript technically sound, and do the data support the conclusions?

The manuscript must describe a technically sound piece of scientific research with data that supports the conclusions. Experiments must have been conducted rigorously, with appropriate controls, replication, and sample sizes. The conclusions must be drawn appropriately based on the data presented.

Reviewer #2: Yes

3. Has the statistical analysis been performed appropriately and rigorously?

Reviewer #2: Yes

4. Have the authors made all data underlying the findings in their manuscript fully available?

The [PLOS Data policy](http://www.plosone.org/static/policies.action#sharing) requires authors to make all data underlying the findings described in their manuscript fully available without restriction, with rare exception (please refer to the Data Availability Statement in the manuscript PDF file). The data should be provided as part of the manuscript or its supporting information, or deposited to a public repository. For example, in addition to summary statistics, the data points behind means, medians and variance measures should be available. If there are restrictions on publicly sharing data—e.g. participant privacy or use of data from a third party—those must be specified.

Reviewer #2: No

Author response:

Done, the following text was added:

"The relevant accession numbers used to access to our data are available on the following links https://www.ncbi.nlm.nih.gov/sra/PRJNA791601 (for all data)."

Please refer to page 8 lines 13 and 14

5. Is the manuscript presented in an intelligible fashion and written in standard English?

PLOS ONE does not copyedit accepted manuscripts, so the language in submitted articles must be clear, correct, and unambiguous. Any typographical or grammatical errors should be corrected at revision, so please note any specific errors here.

Reviewer #2: Yes

6. Review Comments to the Author

Please use the space provided to explain your answers to the questions above. You may also include additional comments for the author, including concerns about dual publication, research ethics, or publication ethics. (Please upload your review as an attachment if it exceeds 20,000 characters)

Reviewer #2: Even though all the comments have been responded but i can't see the file with the changes in different colored text

Author response:

All changes are red colored in the text

7. PLOS authors have the option to publish the peer review history of their article ([what does this mean?](https://journals.plos.org/plosone/s/editorial-and-peer-review-process#loc-peer-review-history)). If published, this will include your full peer review and any attached files.

If you choose “no”, your identity will remain anonymous but your review may still be made public.

**Do you want your identity to be public for this peer review?** For information about this choice, including consent withdrawal, please see our [Privacy Policy](https://www.plos.org/privacy-policy).

Reviewer #2: **Yes:** Muhammad Imran Rashid

**Journal Requirements:**

1. Please review your reference list to ensure that it is complete and correct. If you have cited papers that have been retracted, please include the rationale for doing so in the manuscript text, or remove these references and replace them with relevant current references. Any changes to the reference list should be mentioned in the rebuttal letter that accompanies your revised manuscript. If you need to cite a retracted article, indicate the article’s retracted status in the References list and also include a citation and full reference for the retraction notice.

2. While revising your submission, please upload your figure files to the Preflight Analysis and Conversion Engine (PACE) digital diagnostic tool, <https://pacev2.apexcovantage.com/>. PACE helps ensure that figures meet PLOS requirements. To use PACE, you must first register as a user. Registration is free. Then, login and navigate to the UPLOAD tab, where you will find detailed instructions on how to use the tool. If you encounter any issues or have any questions when using PACE, please email PLOS at [figures@plos.org](mailto:figures@plos.org). Please note that Supporting Information files do not need this step.

**Author response**

1. The reference list was revised. The citation number 50 was added in the citation list, please refer to page 20.

"AL-Hosary A, Răileanu C, Tauchmann O, Fischer S, Nijhof AM, Silaghi C. Tick species identification and molecular detection of tick-borne pathogens in blood and ticks collected from cattle in Egypt. Ticks Tick Borne Dis. 2021;12. doi:10.1016/j.ttbdis.2021.101676"

2. All figure files were uploaded to the Preflight Analysis and Conversion Engine (PACE) (tif format).

Thank you again for your consideration of our revised manuscript.

Yours Sincerely,

**Pr Mohamed Aziz Darghouth**

Laboratoire de Parasitologie, Institution de la Recherche et de l'Enseignement Supérieur Agricoles and Univ. Manouba,

École Nationale de Médecine Vétérinaire de Sidi Thabet, Tunisia

E-mail: darghouth@iresa.tn Phone : +216 98 221 849
